# Successful endovascular stenting of a bleeding external iliac vein mycotic aneurysm in an oncologic patient: a case report

**DOI:** 10.1186/s42155-021-00240-8

**Published:** 2021-06-16

**Authors:** Rupal S. Parikh, Shiyi Li, Christopher Shackles, Tamim Khaddash

**Affiliations:** 1 Department of Interventional Radiology, Hospital of the University of Pennsylvania, 3400 Spruce St, Philadelphia, PA 19104 USA; 2 Perelman School of Medicine, University of Pennsylvania, Philadelphia, PA USA; 3 Interventional Institute at HolyName Medical Center, HolyName Medical Center, Teaneck, NJ USA

**Keywords:** Hemorrhage, Endovascular treatment, Stenting, Venous intervention, Venous aneurysm, Mycotic

## Abstract

**Background:**

Mycotic aneurysms are rare vascular lesions, occurring in 0.6–2% of arterial aneurysms but with no reported venous cases. Venous aneurysms unrelated to an underlying infectious process have been previously described and are typically surgically repaired due to risk of thromboembolic events.

**Case presentation:**

This case reports a bleeding external iliac vein mycotic aneurysm secondary to erosion of a chronic pelvic abscess, successfully treated with endovascular stenting, in an oncologic patient without alternative therapeutic options.

**Conclusion:**

Venous aneurysms are uncommon vascular lesions which have historically been treated with open surgical repair. Given the lower degree of procedural morbidity, endovascular management of these lesions may be an effective option in the appropriate setting, particularly as a last resort in patients without surgical treatment options.

## Background

Arising either from vessel wall damage from a primary infection or secondary infection of a preexisting aneurysm, mycotic aneurysms occur in approximately 0.6–2% of arterial aneurysms (Patel et al., 2019). Given the significant morbidity and mortality associated with mycotic arterial aneurysms, standard of care consists of aggressive antibiotic therapy and surgical debridement/reconstruction (Kim, 2010; Sörelius et al., 2019). With recent advancements however, minimally-invasive management of mycotic arterial aneurysms is becoming an increasingly more common treatment option (Sörelius et al., 2014; Kan et al., 2007). Furthermore, compared to arterial aneurysms, venous aneurysms are rare and more indolent in presentation. Due to the risk of thromboembolic events, recent literature supports treatment of lower extremity deep venous aneurysms, particularly popliteal vein aneurysms (Patel et al., 2019; Teter et al., 2018). While isolated cases of iliac artery mycotic aneurysms are known, no cases involving the iliac vein have been reported and thus management guidelines for these patients are limited (Salam et al., 2017; Brant-Zawadzki et al., 2007; Chandler et al., 2017). This case reports a bleeding external iliac vein (EIV) mycotic aneurysm secondary to erosion of a pelvic abscess into the EIV, which was successfully repaired with endovascular stenting, in a poor surgical candidate with life-saving intent.

## Case presentation

**Fig. 1:**
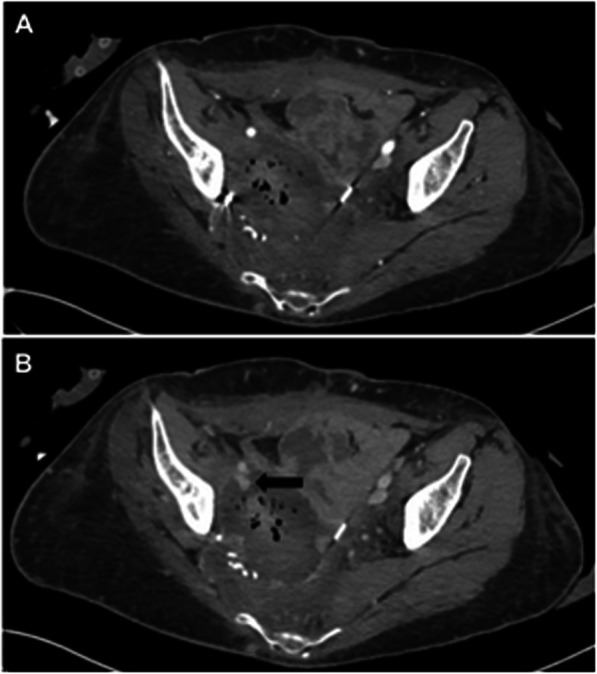
Arterial (**A**) and venous (**B**) phase imaging of CT-abdomen/pelvis demonstrates interval development of venous phase hyperattenuation in the chronic pelvic collection with locules of air, adjacent to a new sac-like dilation arising from the right EIV (black arrow), concerning for vascular erosion by the chronic pelvic abscess

A 34-year-old female with a history of stage IIB cervical squamous cell carcinoma status post pelvic exenteration and external beam radiation, complicated by a pelvic abscess and chronic drainage catheter with prior erosion into the right internal iliac artery status post embolization 1 year prior, was referred to Interventional Radiology (IR) for management of bleeding around and into the pelvic drain. Laboratory evaluation was significant for an acute 5.1 g/dL and 15% decrease in hemoglobin and hematocrit, respectively, compared to the day prior. Multiphasic computed tomography (CT) of the abdomen/pelvis demonstrated interval development of venous phase hyperattenuation in the chronic pelvic collection, adjacent to a new sac-like dilation of the right EIV, concerning for vascular erosion by the chronic pelvic abscess (Fig. 1A and B). The patient was transfused with two units of packed red blood cells with an appropriate increase in hemoglobin/hematocrit. The patient remained hemodynamically stable and was continued on her outpatient intravenous (IV) antibiotic regimen (daptomycin, ceftazidime-avibactam, and metronidazole) for recent *Clostridium* and vancomycin-sensitive enterococcal bacteremia and chronic pelvic abscess per Infectious Disease (ID) recommendations. Given poor surgical candidacy in the setting of extensive pelvic surgery and prior radiation, a multi-disciplinary decision was made to proceed with right external iliac venogram/arteriogram and possible stenting versus embolization.

### Procedure

**Fig. 2:**
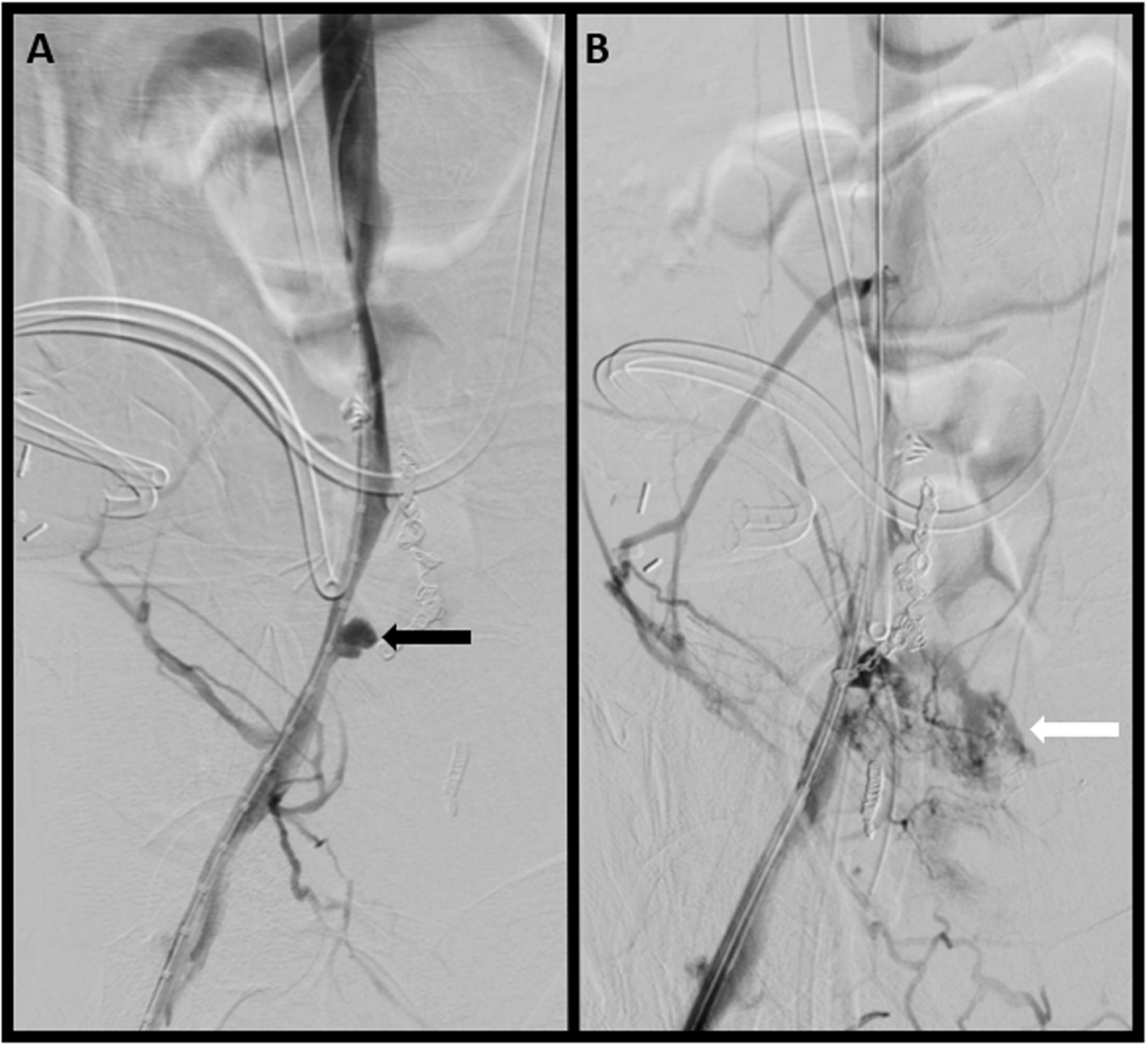
Initial venogram (**A**) of the right EIV demonstrates a focal sac-like outpouching arising from the right EIV (black arrow). Subsequent venogram (**B**) depicts contrast extravasation (white arrow) corresponding to the area of suspected erosion and consequent irregularity of the right EIV seen on cross sectional imaging

Pre-procedure coagulation panel and complete blood count demonstrated normal PT/INR and platelets of 10.5/0.9 and 325, respectively. General anesthesia was administered by a dedicated anesthesia team. The right common femoral vein (CFV) was accessed using standard micropuncture technique. Digital subtraction venography (DSV) of the right CFV to the level of the infrarenal IVC was performed through a transitional sheath, which demonstrated a focal sac-like outpouching arising from the right EIV with eventual contrast extravasation, corresponding to the area of suspected erosion and consequent irregularity of the right EIV seen on cross sectional imaging (Fig. 2A).

**Fig. 3:**
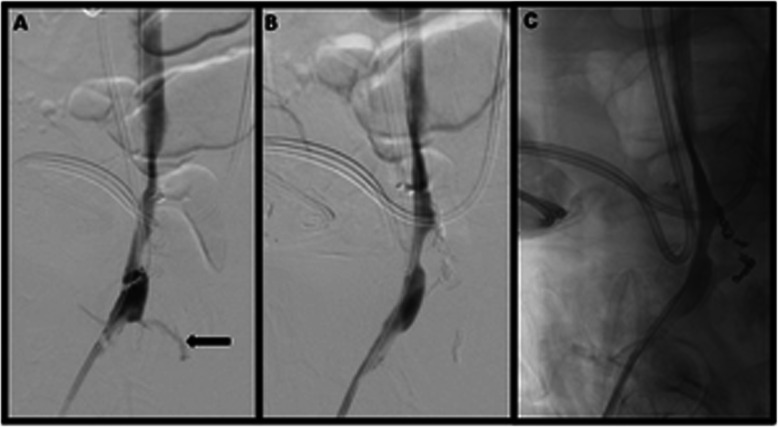
Placement of a 13 mm × 5 cm Viabahn stent graft into the EIV, postdilated to 14 mm, though with persistent contrast extravasation (**A**, black arrow). Placement of an additional 11 mm × 10 cm Viabahn stent graft into the EIV, postdilated to 10 mm, with no persistent extravasation on subtracted and (**B**) unsubtracted (**C**) images

The transitional sheath was then exchanged for a 13 cm long, 12 French (Fr) vascular sheath and a marking catheter was advanced into the right EIV. Based on standard iliac vein averages, a 13 mm × 5 cm Viabahn stent graft was advanced to the targeted area of stenting, and DSV was performed prior to deployment, demonstrating contrast extravasation associated with the pseudoaneurysm (Fig. 2B). The stent was immediately deployed into the EIV at the level of the mycotic venous aneurysm and postdilated to 14 mm (oversized by 1 mm). Post deployment DSV demonstrated an area of contrast extravasation (Fig. 3A), along the inferior margin of the newly deployed stent, concerning for venous rupture into the abscess cavity, likely related to tissue friability from chronic infection. An additional 11 mm × 10 cm Viabahn stent graft was placed through the prior stent to exclude the area of rupture and postdilated to 10 mm without oversizing due to initial venous rupture. Completion DSV demonstrated no evidence of persistent hemorrhage, with brisk inline flow throughout the stent complex into the IVC (Fig. 3B and C). The sheath was removed and manual pressure was maintained until hemostasis was achieved.

**Fig. 4:**
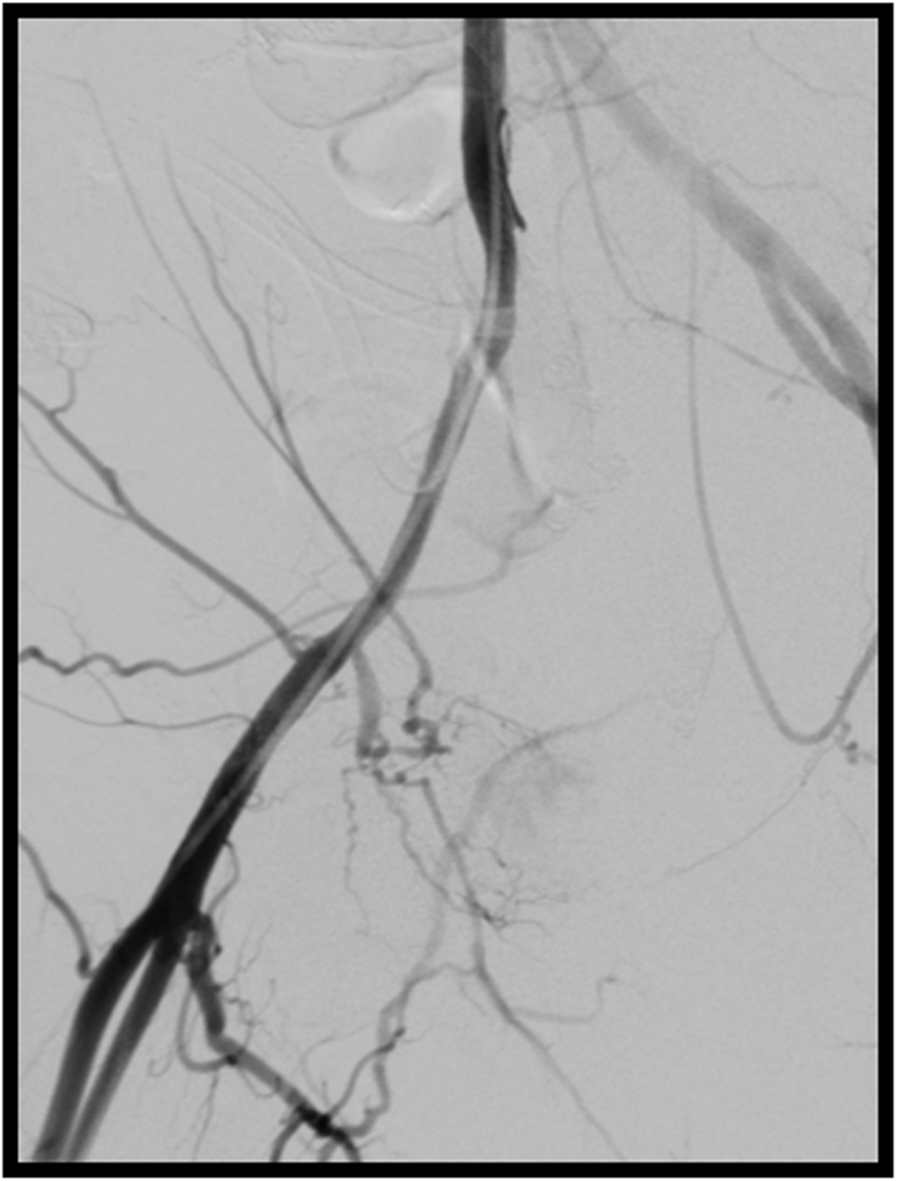
Right external iliac arteriography demonstrates no contrast extravasation to suggest superimposed arterial injury

To exclude any superimposed arterial bleeding in this patient with prior hemorrhage related to right internal iliac artery erosion, the decision was made to study the ipsilateral arterial system. The right common femoral artery (CFA) was then accessed using standard micropuncture technique. A 5 Fr straight flush catheter was advanced “bareback” without a sheath with its tip in the proximal right common iliac artery and digital subtraction arteriography (DSA) of the right external iliac artery was performed in multiple projections with no contrast extravasation to suggest superimposed arterial injury (Fig. 4).

### Post-procedure course

The patient tolerated the procedure well and remained hemodynamically stable throughout her hospital course. Given recent episode of bleeding and risk of erosion into additional branch vessels in the setting of a chronic infection, the decision was made to hold off on anticoagulation. On post-procedure day 2, she was discharged to home on a one-week course of IV antibiotics (regimen described above) with ID follow-up.

**Fig. 5:**
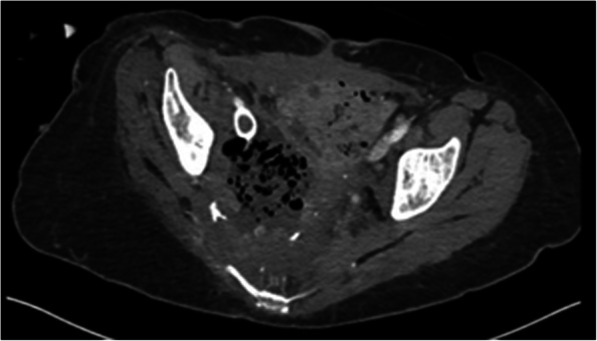
Venous phase CT abdomen/pelvis performed one-month post-procedure demonstrates occlusive right femoral venous thrombosis extending through the stent to the level of the common iliac vein

One month post-procedure, the patient presented to the emergency department with right thigh swelling and pelvic pain. Contrast-enhanced CT abdomen/pelvis demonstrated new occlusive right femoral venous thrombosis extending through the stent to the level of the common iliac vein (Fig. 5). Given concern for superinfection of the occlusive thrombus and increased procedural risk of septic pulmonary emboli, recanalization of the stent was considered high risk and the patient was started on therapeutic anticoagulation with a heparin drip which was transitioned to enoxaparin. She subsequently developed persistent hematuria, at which time a left femoral approach convertible IVC filter was placed and therapeutic anticoagulation was discontinued with resolution of hematuria.

## Conclusions

This case reports a bleeding mycotic venous aneurysm secondary to erosion of a chronic pelvic abscess into the EIV, successfully treated with endovascular stenting. Venous aneurysms are rare vascular lesions with limited published management guidelines. While imaging surveillance is recommended for thoracic and upper extremity aneurysms, open surgical repair, such as aneurysmectomy, venorrhaphy, and end-to-end anastomosis, is indicated for lower extremity aneurysms due to a higher risk of thromboembolic events (Teter et al., 2018). Only a few cases of endovascular treatment of mycotic aneurysms have been reported (Chandler et al., 2017; San Norberto et al., 2010). Furthermore, venography and endovascular management of mycotic venous aneurysms have not been reported. The iliac veins, in particular, may be more amenable to stenting given their deep and relatively fixed locations. The present report demonstrates that endovascular stenting is a safe and effective option in patients with EIV mycotic aneurysms, especially as a last resort in oncologic patients with complex surgical histories, predisposing to adhesive disease. While the result was not durable in terms of long-term patency, life threatening hemorrhage was prevented and the patient’s life was extended.

Venous aneurysms are uncommon vascular lesions which have historically been treated with open surgical repair. The present report demonstrates that endovascular stenting is an effective option in patients with mycotic EIV aneurysms, especially to prevent life threatening hemorrhage in patients who are poor surgical candidates.

## Data Availability

Not applicable.

## Abbreviations

EIV: External iliac vein
IR: Interventional radiology
CT: Computed tomography
IV: Intravenous
CFV: Common femoral vein
DSV: Digital subtraction venography
Fr: French
CFA: Common femoral artery
